# Genetic and Structural Variation in the O-Antigen of Salmonella enterica Serovar Typhimurium Isolates Causing Bloodstream Infections in the Democratic Republic of the Congo

**DOI:** 10.1128/mbio.00374-22

**Published:** 2022-07-18

**Authors:** Sandra Van Puyvelde, Gianmarco Gasperini, Michael Biggel, Marie-France Phoba, Maria Michelina Raso, Tessa de Block, Leen N. Vanheer, Stijn Deborggraeve, Olivier Vandenberg, Nicholas Thomson, Neil Ravenscroft, Calman A. Maclennan, Barbara Bellich, Paola Cescutti, Gordon Dougan, Jan Jacobs, Octavie Lunguya, Francesca Micoli

**Affiliations:** a Cambridge Institute of Therapeutic Immunology and Infectious Disease, Department of Medicine, University of Cambridge, Cambridge, United Kingdom; b Laboratory of Medical Microbiology, Vaccine and Infectious Disease Institute, University of Antwerp, Antwerp, Belgium; c Wellcome Trust Sanger Institute, Hinxton, United Kingdom; d Institute of Tropical Medicine, Antwerp, Belgium; e GSK Vaccines Institute for Global Health, Siena, Italy; f Department of Microbiology, Institut National de Recherche Biomédicale, Kinshasa, Democratic Republic of the Congo; g Department of Medical Biology, University Teaching Hospital of Kinshasa, Kinshasa, Democratic Republic of the Congo; h Clinical Research and Innovation Unit, Laboratoire Hospitalier Universitaire de Bruxelles-Universitair Laboratorium Brussel, Université Libre de Bruxelles, Brussels, Belgium; i Centre for Environmental Health and Occupational Health, School of Public Health, Université Libre de Bruxelles, Brussels, Belgium; j Division of Infection and Immunity, Faculty of Medical Sciences, University College London, London, United Kingdom; k London School of Hygiene and Tropical Medicine, London, United Kingdom; l Department of Chemistry, University of Cape Town, Rondebosch, South Africa; m Jenner Institute, Nuffield Department of Medicine, University of Oxford, Oxford, United Kingdom; n Department of Life Sciences, University of Trieste, Trieste, Italy; o Department of Microbiology, Immunology and Transplantation, KU Leuven, Leuven, Belgium; Pasteur Institute

**Keywords:** Democratic Republic of the Congo, O-antigen, *Salmonella* Typhimurium, genomics, iNTS, surface structures, vaccines, whole-genome sequencing

## Abstract

Salmonella enterica serovar Typhimurium causes a devastating burden of invasive disease in sub-Saharan Africa with high levels of antimicrobial resistance. No licensed vaccine is available, but O-antigen-based candidates are in development, as the O-antigen moiety of lipopolysaccharides is the principal target of protective immunity. The vaccines under development are designed based on isolates with O-antigen O-acetylated at position C-2 of abequose, giving the O:5 antigen. Serotyping data on recent Salmonella Typhimurium clinical isolates from the Democratic Republic of the Congo (DRC), however, indicate increasing levels of isolates without O:5. The importance and distribution of this loss of O:5 antigen in the population as well as the genetic mechanism responsible for the loss and chemical characteristics of the O-antigen are poorly understood. In this study, we Illumina whole-genome sequenced 354 Salmonella Typhimurium isolates from the DRC, which were isolated between 2002 and 2017. We used genomics and phylogenetics combined with chemical approaches (^1^H nuclear magnetic resonance [NMR], high-performance anion-exchange chromatography with pulsed amperometric detection [HPAEC-PAD], high-performance liquid chromatography–PAD [HPLC-PAD], and HPLC-size exclusion chromatography [HPLC-SEC]) to characterize the O-antigen features within the bacterial population. We observed convergent evolution toward the loss of the O:5 epitope predominantly caused by recombination events in a single gene, the *O*-acetyltransferase gene *oafA*. In addition, we observe further O-antigen variations, including O-acetylation of the rhamnose residue, different levels of glucosylation, and the absence of O-antigen repeating units. Large recombination events underlying O-antigen variation were resolved using long-read MinION sequencing. Our study suggests evolutionary pressure toward O-antigen variants in a region where invasive disease by Salmonella Typhimurium is highly endemic. This needs to be taken into account when developing O-antigen-based vaccines, as it might impact the breadth of coverage in such regions.

## INTRODUCTION

The bacterium Salmonella enterica serovar Typhimurium is globally known as a common cause of gastrointestinal infections. However, *S*. Typhimurium is also responsible for an urgent health burden in sub-Saharan Africa by causing severe bloodstream infections. Two-thirds of invasive nontyphoidal Salmonella (iNTS) infections are caused by S. enterica serovar Typhimurium, and one-third are caused by S. enterica serovar Enteritidis, jointly responsible for over 500,000 bloodstream infections per year in sub-Saharan Africa (1). The fatality rate of iNTS infections can be extremely high and has been estimated to be up to 20% (2). iNTS infections disproportionally affect children under 5 years of age, with malnutrition and coinfection with malaria and HIV as major risk factors (2).

*S*. Typhimurium is identified upon serotyping, presenting a specific combination of O-antigen (OAg) and H-antigen compounds. *S*. Typhimurium further has multiple sequence types (STs), with ST19, -34, and -313 being the most reported. Bloodstream infections observed in sub-Saharan Africa are specifically associated with ST313, which is comprised of lineage I and lineage II that sequentially spread in this region (3). ST313 lineage I was found mostly in East African countries, whereas ST313 lineage II was identified across sub-Saharan Africa and was found to be dominant in recent studies (3–6). A higher diversity of lineages is seen in Kenya, where ST19 isolates were found to cause a substantial proportion of bloodstream infections, similar to ST313 isolates from lineages I and II (7).

Antimicrobial resistance (AMR) is high and further increasing, which threatens effective treatment (8). In the Democratic Republic of the Congo (DRC), an extensively drug-resistant (XDR) sublineage outbreak has recently been identified, which remained susceptible to only one available antibiotic, ciprofloxacin, and two isolates were found to be pan-drug resistant (PDR) (4), i.e., resistant to all available antibiotics.

Alternative intervention strategies for iNTS are urgently needed, especially in regions threatened by a high prevalence and/or high AMR levels. No licensed vaccine is currently available, but different candidates are in development (9). The OAg moiety of bacterial cell surface lipopolysaccharides (LPSs) is the principal target of protective immunity (10–14). OAg-based vaccines targeting iNTS, including glycoconjugate vaccines (LPS-derived OAg linked to carrier proteins) and generalized modules for membrane antigens (GMMA) (outer membrane vesicles consisting of surface polysaccharides and outer membrane proteins), are in development and have been shown to provide protection in *in vitro* and *in vivo* models (11, 15–19).

The *S*. Typhimurium OAg is composed of repeating units containing the monosaccharides mannose (Man), rhamnose (Rha), and galactose (Gal) (O:12 specificity), which can be variably glucosylated at galactose, through (1→6) or (1→4) linkages conferring O:1 and O:12_2_ specificities, respectively (20). The immunodominant dideoxyhexose abequose (Abe) linked α-(1→3) to mannose confers O:4 specificity. The Abe and Rha sugars can be further O-acetylated (21). O:5 specificity is conferred by O-acetylation at position C-2 of Abe (21).

10.1128/mbio.00374-22.3 TABLE S1 List of all Salmonella Typhimurium isolates. For all isolates from this data set, the sample name, strain name, province in the Democratic Republic of Congo, year of isolation, O5 serotype, whether these were selected for chemical analysis, and conservation of the *oafA* gene are indicated. Download Table S1, XLSX file, 0.02 MB. Copyright © 2022 Van Puyvelde et al. 2022 Van Puyvelde et al. https://creativecommons.org/licenses/by/4.0/ This content is distributed under the terms of the Creative Commons Attribution 4.0 International license.

*S*. Typhimurium O:5-negative (O:5^−^) isolates were first discovered by F. Kauffman in 1934 and classified as the “Copenhagen *varietas*.” These O:5^−^ variants were reported in pigeons, heifers, cattle, and swine and occasionally in dogs and cats, while cases in humans have been extremely rare (22, 23). However, O:5 specificity is not part of routine *S*. Typhimurium serological identification. Data collected during 10 years of surveillance in the DRC for which the O:5 specificity was studied indicate that there is a large proportion of *S*. Typhimurium clinical isolates lacking O:5, estimated to be 45% of all clinical isolates in 2017 (24). However, the vaccines under development are designed based on O:5-containing (O:5^+^) isolates.

In this work, we used a combined genomics and chemical approach to study this OAg variation in the bacterial population of *S*. Typhimurium causing bloodstream infections in the DRC.

## RESULTS

### Convergent loss of O:5 specificity in the *S*. Typhimurium population is caused by recombination in *oafA*.

O:5^−^ invasive *S*. Typhimurium isolates were increasingly observed in the DRC (8). We observed that within a selection of these isolates, the O:5 phenotype was stable after subculturing and over time, which would not be expected if this were a bistable phenotype. This suggests that the loss of the O:5 specificity in the DRC isolates has a genetic cause in the O:5^−^ isolates and is not a result of variable expression in the *S*. Typhimurium population.

**FIG 1:**
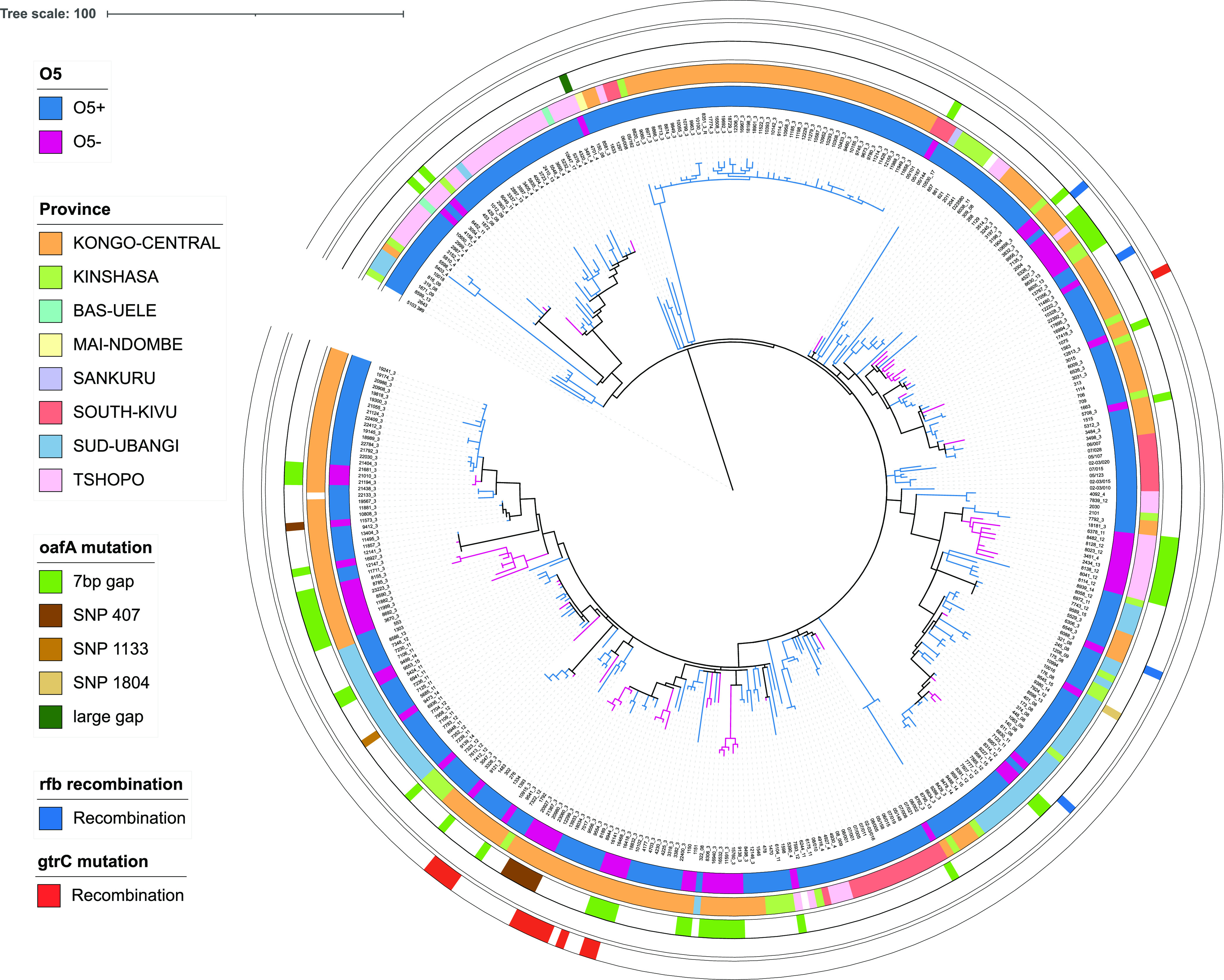
Phylogenetic tree of invasive Salmonella Typhimurium isolates from the Democratic Republic of the Congo (DRC) indicating the presence and absence of the O:5 antigen phenotype. The maximum likelihood phylogenetic tree is based on the 354 genome sequences from this study (summarized in Table S1 in the supplemental material). Sequencing reads were mapped to *S*. Typhimurium ST313 lineage II reference strain D23580 (50). The tree is based on 2,131 chromosomal SNPs. Metadata are visualized on the concentric rings according to the key as follows, from the inside to the outside; O:5 phenotype (ring 1); province of isolation (ring 2); and mutation in the *oafA* gene, *rfb* operon, and *gtrC* gene (rings 3 to 5). Branches are colored by the O:5 phenotype. Isolate DT2 is included as the outgroup to root the tree. Branch lengths represent the number of SNPs, as indicated by the scale bar.

To identify the genetic cause, a total of 354 *S*. Typhimurium isolates with accompanying metadata and serotype data were whole-genome sequenced. All isolates were identified as closely related ST313 lineage II isolates by building a core-genome phylogenetic tree (Fig. 1). The 71 O:5^−^ *S*. Typhimurium isolates were found across ST313 lineage II. When the O:5 phenotype was marked across the phylogeny, it showed at least 30 independent changes from O:5^+^ to O:5^−^. Multiple monophyletic branches with many independently arisen O:5^−^ isolates were evident. These isolates appear sporadically throughout the tree, implying the frequent local loss and circulation of O:5^−^ isolates in the DRC. While the different clusters of O:5^−^ isolates predominantly remained within a province, some showed spread across the DRC. Six of these clusters were confined to the Kongo-Central province, one was confined to Sud-Ubangi, another cluster was found in both Tshopo and Sud-Ubangi, and one cluster was found in Kongo-Central and Kinshasa.

**FIG 2:**
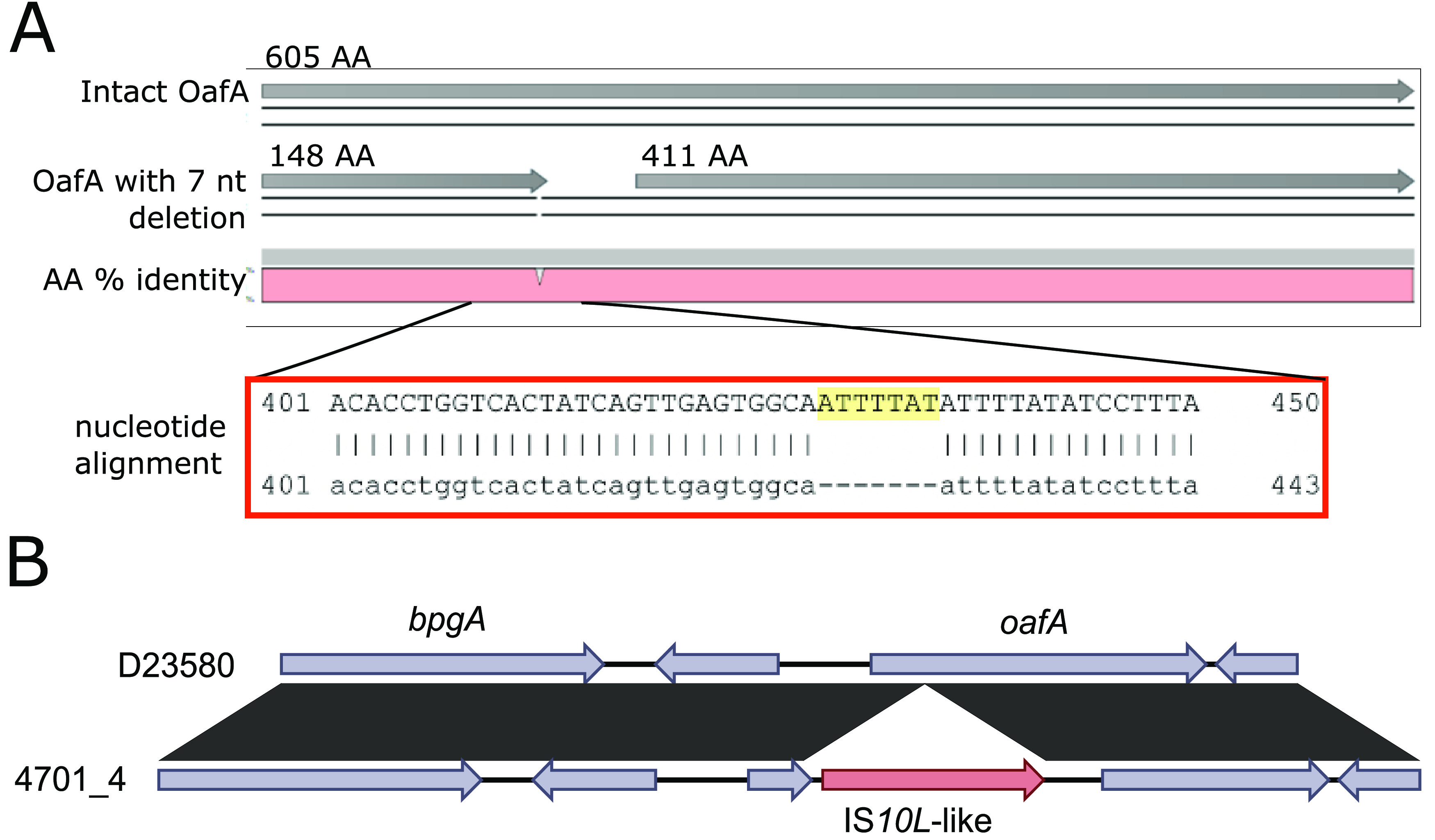
Mutational events in the *oafA* gene associated with the loss of the O:5 phenotype. (A) Pairwise amino acid (AA) assembly of an intact OafA protein and the OafA protein sequence of an isolate showing a frameshift mutation linked to a 7-nucleotide ATTTTAT deletion in the *oafA* gene. (B) Pairwise comparison of the *oafA* gene sequences of D23580 (O:5^+^) and 4701/4 (O:5^−^) presenting an insertion of an IS*4* family transposase into *oafA*, as resolved through MinION long-read sequencing.

A pangenomic analysis combined with a genome-wide association study (GWAS) identified the presence of the *O*-acetyltransferase gene *oafA* (also named *oatA*, *yrhL*, or STM2232) as being most significantly associated with the loss of the O:5 phenotype (Bonferroni-corrected *P* value of 1.65e−41). The genes neighboring *oafA* are the *umuD* gene and a predicted phage tail fiber assembly gene, which was previously shown to mediate OAg abequose O-acetylation and, hence, O:5 specificity in *S*. Typhimurium (25, 26). By further investigation of single nucleotide polymorphisms (SNPs) and variations in the genome assemblies, the *oafA* gene was found to be intact in all O:5^+^ isolates (*n* = 283), while truncations in the *oafA* gene were observed in the O:5^−^ isolates. The majority of the O:5^−^ isolates showed the same 7-nucleotide (nt) deletion at position 429, resulting in a frameshift mutation that breaks the *oafA* gene into two smaller fragments (*n* = 53). A 7-nt sequence (ATTTTAT) is present as repeating units in the O:5^+^ isolate genomes, but one of the repeating units has been lost in the O:5^−^ isolates (Fig. 2A). Six isolates forming one monophyletic branch showed a 407G-A SNP in *oafA* resulting in a premature stop codon (TGG [W] to TAG *amber* [stop] codon), one isolate showed an 1133G-A SNP in *oafA* resulting in a premature stop codon (TGG [W] to TAG *amber* [stop] codon), and one isolate showed an 1804G-T SNP in *oafA*, again resulting in a premature stop codon (GAA [E] to TAA *ochre* [stop] codon). Finally, the *oafA* gene in isolate 4701/4 was truncated by the insertion of an IS*4* transposase (Fig. 2B).

Our data thus show the repeated evolutionary loss of O:5^−^ specificity in the *S*. Typhimurium population predominantly driven by an independent deletion of a 7-nt sequence within *oafA* but also with other isolates showing alternative mutational events acting at the level of transcription or that truncate or disrupt the function of the translated OafA protein product (Fig. 1).

### O:5^−^ isolates lack O-acetylation of Abe or the full OAg.

Twenty-five invasive *S*. Typhimurium isolates were chosen for full chemical characterization, including 10 O:5^−^ isolates presenting *oafA* mutations, 4 O:5^−^ isolates with intact *oafA* genes, and 11 O:5^+^ isolates with intact *oafA* genes (Table 1; see also Fig. S1 in the supplemental material). These isolates were chosen to reflect (i) the different types of *oafA* mutations observed, (ii) the genetic diversity in the *S*. Typhimurium phylogeny, and (iii) the spatiotemporal diversity in the overall collection. These isolates were analyzed for OAg production levels, structural modifications (O-acetylation position and glucosylation level), and the OAg molecular size distribution. The results obtained are summarized in Table 1.

**TABLE 1:**
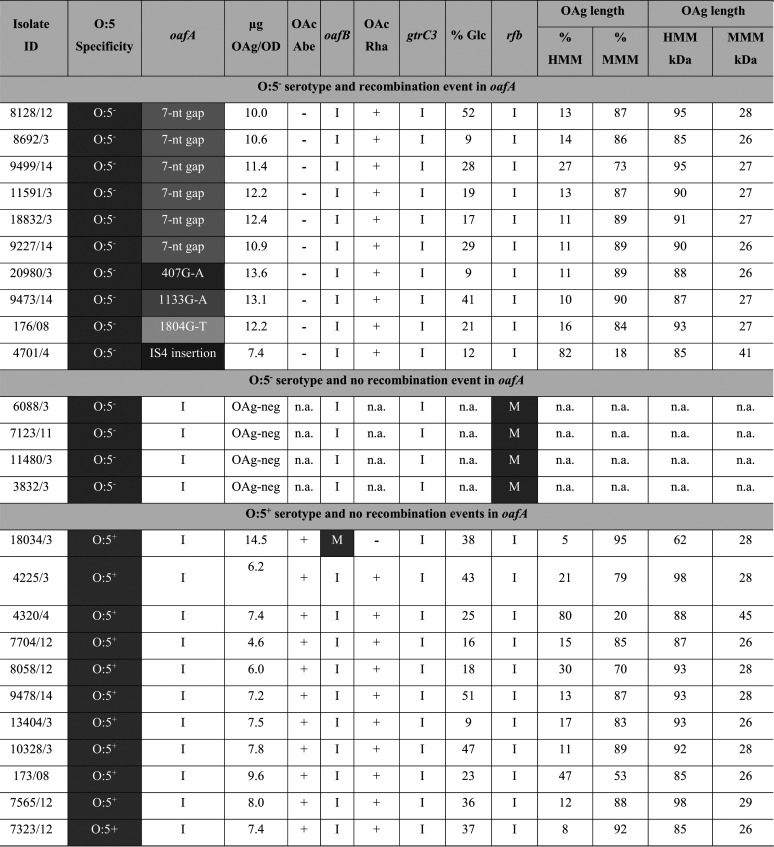
Overview of genomic and physicochemical characterization of isolates studied^*a*^

a For each isolate, the isolate identifier is given, along with the O:5 phenotype as determined upon isolation and collection; the conservation of the *oafA*, *gtrC2*, *gtrC3*, and *rfb* genes as determined by Illumina and MinION sequencing analyses; the OAg production levels (micrograms of OAg/OD unit by HPAEC-PAD after OAg isolation); O-acetylation (OAc) of the abequose (Abe) and rhamnose (Rha) residues as determined by ^1^H NMR; the percentage of glucosylation (Glc) as determined by HPAEC-PAD; and the OAg length by HPLC-SEC (average size determined by using dextrans as standards and percentage of populations at different relative sizes as determined by dRI areas of the peaks). HMM, high-molecular-mass OAg; MMM, medium-molecular-mass OAg; I, intact; M, mutated; n.a., not applicable.

10.1128/mbio.00374-22.1 FIG S1 Phylogenetic tree of invasive Salmonella Typhimurium isolates from the Democratic Republic of the Congo (DRC) with the O:5 phenotype. Shown is a maximum likelihood phylogenetic tree based on the 352 genome sequences from this study (summarized in Table S1 in the supplemental material). Sequencing reads were mapped to *S*. Typhimurium ST313 lineage II reference strain D23580 (49). The tree is based on 2,131 chromosomal SNPs. Metadata are visualized on the concentric rings according to the key as follows, from the inside to the outside: O:5 phenotype (ring 1); province of isolation (ring 2); mutation in the *oafA* gene, *rfb* operon, and *gtrC2* gene (rings 3 to 5); and selection of isolates for chemical analysis (ring 6). Branches are colored by the O:5 phenotype. Isolate DT2 is included as the outgroup to root the tree. Branch lengths represent the number of SNPs, as indicated by the scale bar. Download FIG S1, EPS file, 0.6 MB. Copyright © 2022 Van Puyvelde et al. 2022 Van Puyvelde et al. https://creativecommons.org/licenses/by/4.0/ This content is distributed under the terms of the Creative Commons Attribution 4.0 International license.

The O:5^−^ isolates with *oafA* mutations all showed an absence of 2-O-acetylation at the Abe residue, irrespective of the disruption to *oafA*.

**FIG 3:**
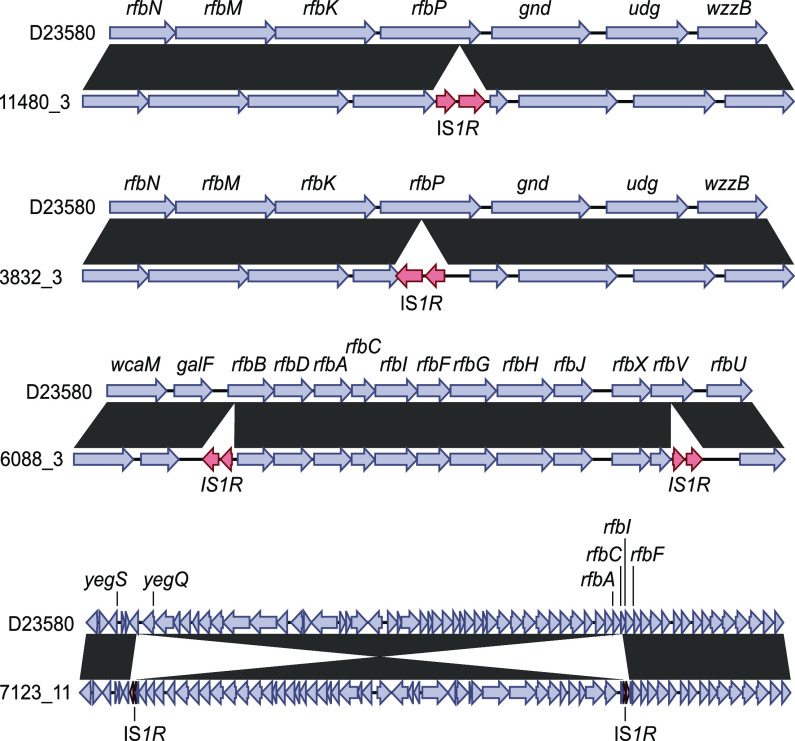
Resolved *rfb* recombinations underlying OAg loss using MinION sequencing. The genome sequences of isolates 11480/3, 3832/3, 6088/3, and 7123/11 are pairwise compared to that of isolate D23580 (GenBank accession number FN424405). Homologous regions between both sequences are colored according to the scale bar. Transposase genes are shown in red. Genes of *S*. Typhimurium D23580 are annotated with the *S*. Typhimurium LT homologs (GenBank accession number AE006468.2).

Characterization of the four O:5^−^ isolates with intact *oafA* genes by flow cytometry and LPS silver staining suggested that these isolates were OAg negative (Fig. S2). The absence of OAg was confirmed by ^1^H nuclear magnetic resonance (NMR) and high-performance anion-exchange chromatography with pulsed amperometric detection (HPAEC-PAD). These four OAg-negative isolates possessed transposase insertions within the *rfb* locus, which could be fully resolved by MinION long-read sequencing (Fig. 3). *rfb* (or *wb*) genes encode the OAg biosynthesis of Gram-negative bacteria and include genes involved in nucleotide sugar synthesis and O-unit assembly (27).

10.1128/mbio.00374-22.2 FIG S2 Characterization of O:5^−^ isolates with intact *oafA* genes. Flow cytometry and LPS silver staining were used. Isolates 6088/3 and 7123/11 were O:5^−^ with intact *oafA* isolates and did not present OAg structures. Download FIG S2, TIF file, 0.7 MB. Copyright © 2022 Van Puyvelde et al. 2022 Van Puyvelde et al. https://creativecommons.org/licenses/by/4.0/ This content is distributed under the terms of the Creative Commons Attribution 4.0 International license.

The OAg-negative isolates containing transposase insertions in the *rfb* locus were found as singletons distributed across the phylogeny, with mutational events that suggest that they have arisen multiple times and independently (Fig. 1).

### Further diversity in the *S*. Typhimurium OAg structure due to O-acetylation and glucosylation.

**FIG 4:**
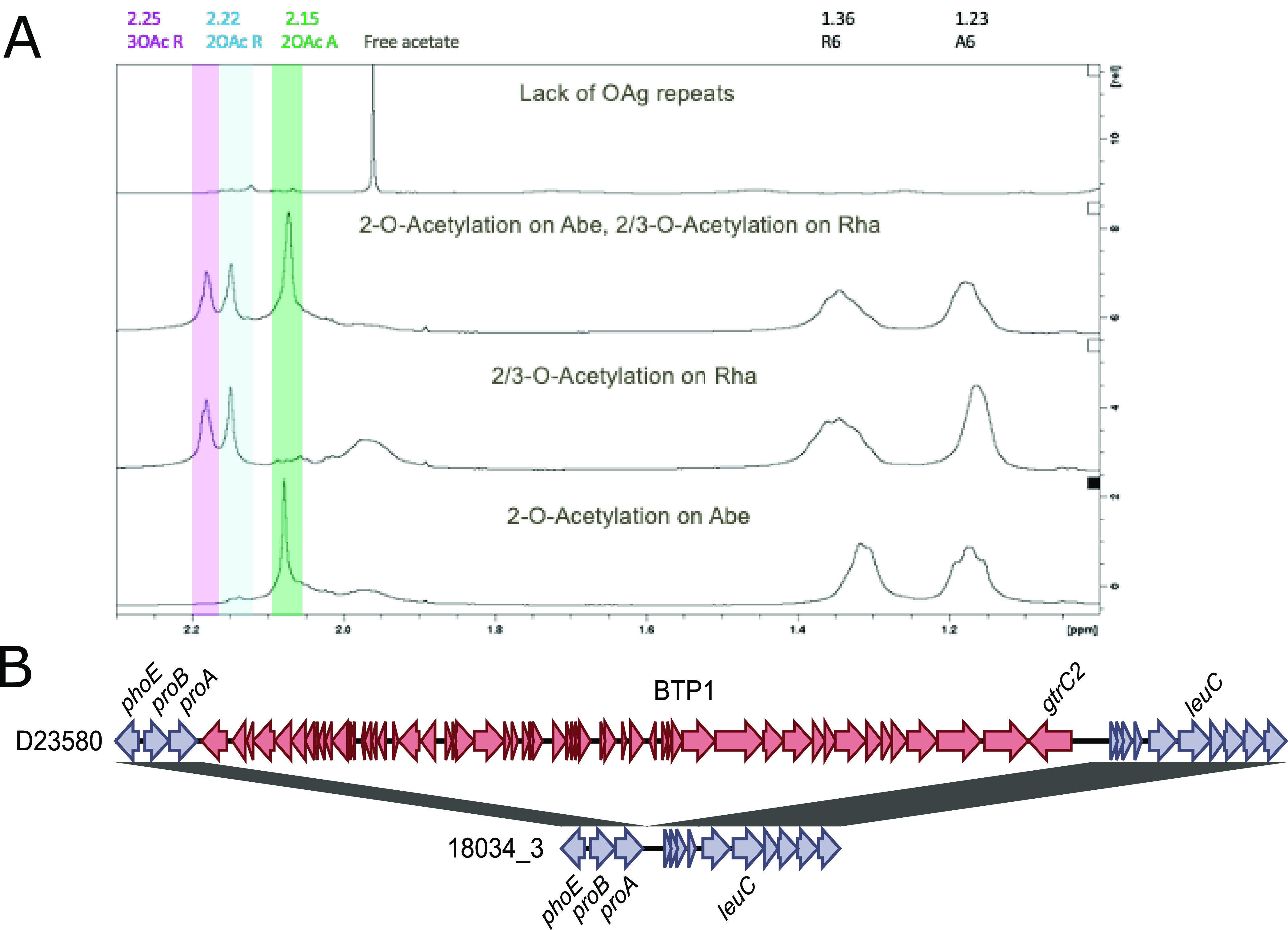
Diverse O-acetylation patterns. (A) ^1^H NMR spectra of OAgs extracted from 4 isolates representative of the variability observed. From top to bottom, the spectra are shown from an isolate that (i) lacks OAg repeats, (ii) is O-acetylated (OAc) on both rhamnose (Rha) and abequose (Abe), (iii) is O-acetylated on Rha only, or (iv) is O-acetylated on Abe only. (B) Resolved *grtC2* recombination underlying the loss of O-acetylation of rhamnose in isolate 18034/3 compared to isolate D23580. Homologous regions between both sequences are colored according to the scale bar.

Our ^1^H NMR spectra showed that in addition to the O-acetylation on C-2 of Abe, all isolates, with the exception of 18034/3, displayed OAg with O-acetylation on C-2 and C-3 of Rha, independently from the O:5 specificity (Fig. 4A and Table 1). The presence of 2/3-OAc (indicating that OAc can be in position 2 or 3 of the rhamnose sugar ring) on Rha has previously been observed for the *S*. Typhimurium ST313 reference isolate D23580 and was acquired through the uptake of the BTP1 prophage (21, 28, 29). The genetic determinant of this OAg modification was formerly named *gtrC2* (or *gtrCc* or *gtrC_BTP1*) by association with GtrABC glycosyltransferase family 2. However, as no functional relationship is present, the gene was recently renamed *oafB*, similar to the name already in use for *oafA* (30). MinION sequencing resolved the full genome of isolate 18034/3 and revealed recombination in which the full region was lost, including *oafB* (Fig. 4B). In the full isolate collection, 13 isolates lacked the *oafB* gene, of which 3 were O:5^−^ and 10 were O:5^+^. These 13 isolates are all related strains originating from Kongo-Central (Fig. S1).

Glucosylation levels varied between 9 and 52% among the different isolates that were chemically characterized (Table 1). All these 25 isolates showed the presence of an intact *gtrC3* gene, responsible for the α1-4 linkage of glucose on galactose in the OAg repeating unit (O:12_2_ specificity). None of these isolates showed the presence of a *gtrC1* gene, responsible for the α1-6 linkage of glucose on galactose in the OAg repeating unit (O:1 specificity). A genetic cause underlying the variability in OAg O-acetylation and glucosylation levels in the characterized isolates could not be identified.

All of the OAg-positive isolates showed a bimodal distribution of the OAg chain length, which is associated with the presence of intact *wzzB* and *fepE* genes (31). The relative amounts of high-molecular-mass (HMM) OAg and medium-molecular-mass (MMM) OAg varied among the different isolates (Table 1). HMM OAg varied between 85 and 98 kDa, with the exception of 1 isolate with no O-acetylation on Rha, having an HMM OAg of 62 kDa. MMM OAg ranged between 26 and 29 kDa, but two isolates had MMM OAgs of higher molecular masses of around 40 kDa.

Interestingly, it was observed that the OAg expression levels were lower in the strains expressing OAg O-acetylated at both Abe and Rha positions (Table 1).

Five OAgs (produced by isolates 18034/3, 8128/12, 8692/3, 13404/3, and 10328/3) differing in the patterns of O-acetylation and levels of glucosylation were selected to be further purified and subjected to more detailed structural characterization by ^1^H NMR and gas-liquid chromatography coupled with mass spectrometry (GLC-MS). ^1^H NMR confirmed the expected pattern and degree of O-acetylation (Table S2). Composition and linkage analyses by GLC-MS confirmed the identity of the expected monosaccharides together with their glycosidic linkages. In particular, it was confirmed that the Gal residue was glucosylated on C-4, and the measured percentages of glucosylation were in agreement with those found by HPAEC-PAD (Table S3).

10.1128/mbio.00374-22.4 TABLE S2 O-acetylation levels of a subset of isolates. For each isolate, the isolate identifier is given, along with the percentages of O-acetylation (OAc) of the abequose (Abe) and rhamnose (Rha) residues as determined by ^1^H NMR. Download Table S2, DOCX file, 0.03 MB. Copyright © 2022 Van Puyvelde et al. 2022 Van Puyvelde et al. https://creativecommons.org/licenses/by/4.0/ This content is distributed under the terms of the Creative Commons Attribution 4.0 International license.

10.1128/mbio.00374-22.5 TABLE S3 Percentage of glucosylation of a subset of isolates. For each isolate, the isolate identifier is given, along with the percentage of glucosylation (Glc) as determined by GLC-MS. Download Table S3, DOCX file, 0.03 MB. Copyright © 2022 Van Puyvelde et al. 2022 Van Puyvelde et al. https://creativecommons.org/licenses/by/4.0/ This content is distributed under the terms of the Creative Commons Attribution 4.0 International license.

### Impact of O:5 specificity on the binding capacity and bactericidal activity of different anti-OAg antibodies.

**FIG 5:**
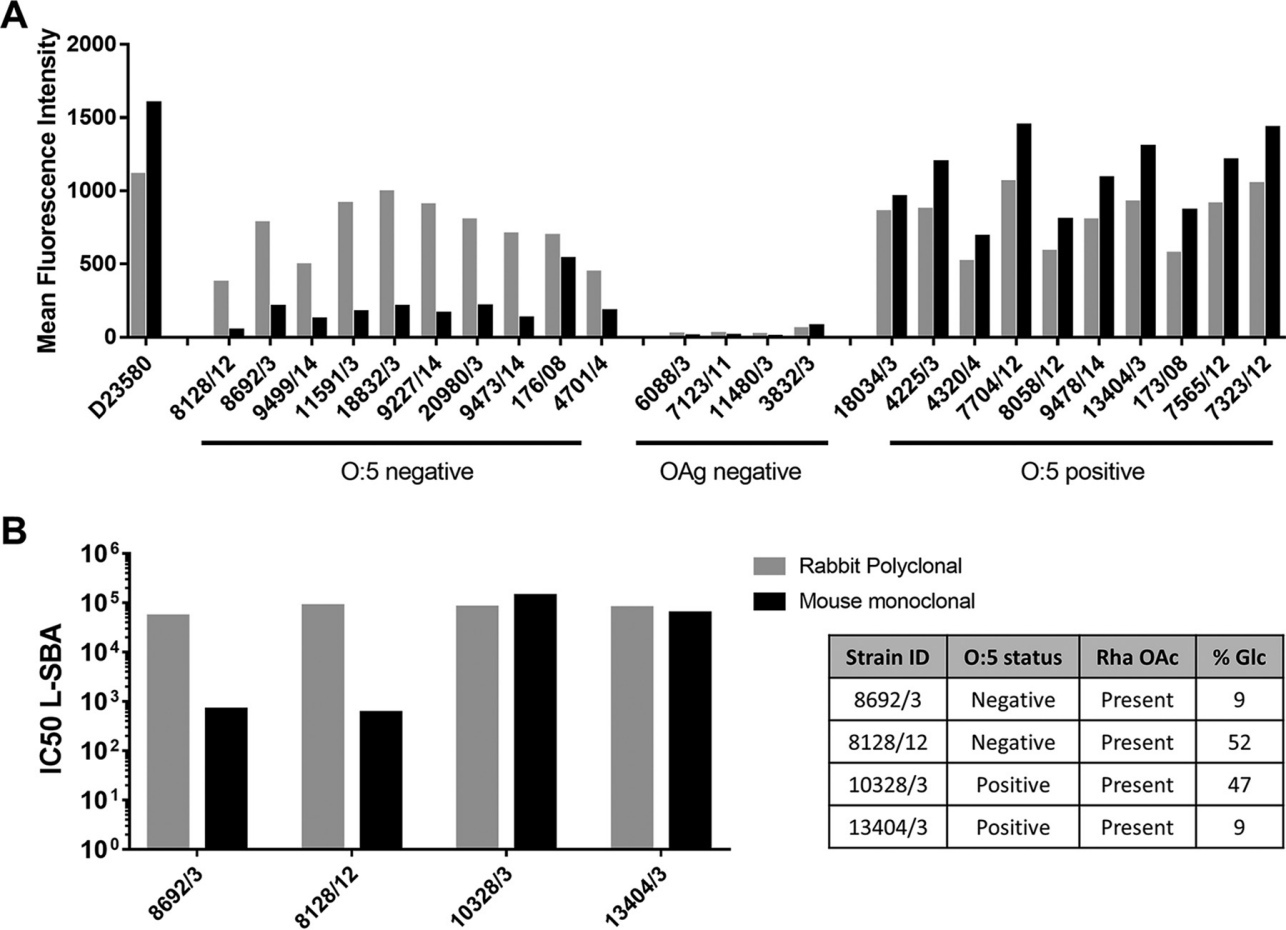
Binding capacity and bactericidal activity of commercial anti-OAg antibodies. (A) FACS analysis of *S*. Typhimurium isolates using a rabbit polyclonal or a mouse monoclonal antibody. The mean fluorescence intensities (MFIs) of 10,000 acquired events are reported. (B) Serum bactericidal activity of commercial antibodies against the selected *S*. Typhimurium strains. IC_50_ values, corresponding to 50% bacterial growth inhibition, are reported.

To investigate whether the observed OAg structural diversity could impact the ability of specific antibodies to bind to the bacterial surface, the panel of 25 selected isolates was analyzed by fluorescence-activated cell sorting (FACS) using commercial anti-OAg antibodies. As a control, *S*. Typhimurium strain D23580 (O:5^+^) was included in the analysis. Interestingly, while a rabbit polyclonal antibody (catalog number 294401; Denka Seiken) showed similar abilities to bind to all tested isolates irrespective of the O:5 status, a mouse monoclonal antibody (catalog number ab8274; Abcam) showed higher specificity for O:5*^+^* isolates (Fig. 5A). The same antibodies were tested in a serum bactericidal assay (SBA) to confirm their ability to trigger complement-mediated killing of isolates 8128/12 (O:5^−^), 8692/3 (O:5^−^), 13404/3 (O:5^+^), and 10328/3 (O:5^+^), also selected based on the different levels of OAg glucosylation (Table 1). Strikingly, while similar killing was observed against all isolates using the rabbit polyclonal serum, significantly less killing was observed against O:5^−^ isolates using the mouse monoclonal antibody (Fig. 5B), independently from OAg glucosylation.

## DISCUSSION

**FIG 6:**
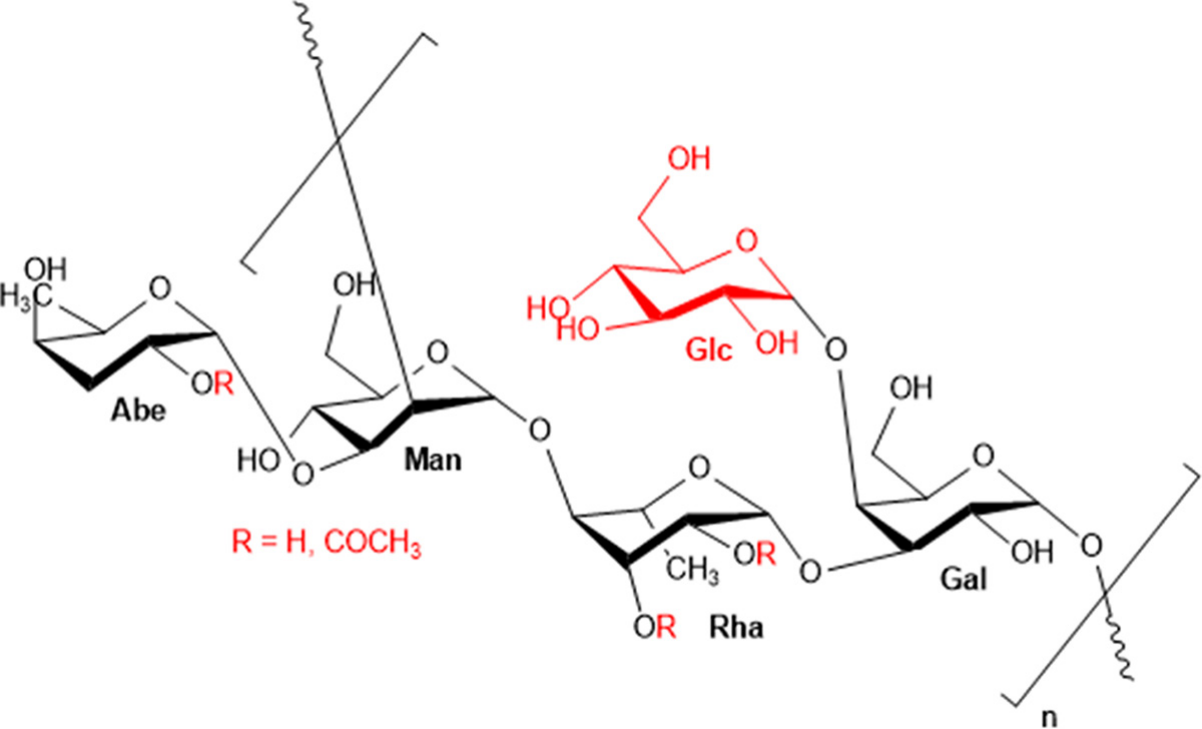
*S*. Typhimurium O-antigen repeating unit. The observed modifications are highlighted in red: O-acetylation on Abe or Rha residues and glucosylation.

We have observed an unexpectedly high level of OAg variation in the *S*. Typhimurium population in the DRC, as summarized in Fig. 6. This variation includes the loss of OAg, Abe or Rha O-acetylation, and differences in glucosylation levels. The absence of O-acetylation of the Abe residue resulted from mutations in *oafA* that explain the O:5^−^ phenotype. Disruption of the production of O:5 LPS was highly prevalent in our *S*. Typhimurium data set, with multiple O:5^−^ isolates being present across the phylogeny in monophyletic lineages. The distribution of the O:5^−^ isolates suggests that these mutations occurred in the DRC itself and that the O:5^−^ isolates possessing these genetic lesions have spread further locally. This suggests that there might be a selective advantage to the loss of O:5^+^ by mutation of *oafA* among invasive *S*. Typhimurium isolates in the DRC.

A lack of O-acetylation of the Rha residue or a loss of the full OAg, however, was observed but at lower rates and is linked to a diverse set of mutational events. As these events were observed in single isolates scattered across the phylogenetic tree, the clinical importance of these variations remains unclear, and these mutations are also likely to be an artifact caused by passage in the laboratory.

Variation in the *S*. Typhimurium OAg can impact infection, as it was previously shown to play a role in the interaction with the host. The length of the OAg affects colitis and impacts the interaction with macrophages and complement (32, 33). Phase-variable chain length has been shown to be a trade-off between virulence and bacteriophage resistance (34), while phase-variable glucosylation acts as a defense against bacteriophages (35). Unfortunately, no tissue samples or clinical details from patients were available to inform on whether these O:5^−^ isolates caused an altered host response or a more severe disease outcome.

Interestingly, a recent study identified *S*. Typhimurium IgA escape mutants in mice that were vaccinated but diseased (36). OAg variants similar to the ones that we identified in the DRC were found among the escape mutants, including a loss of Abe O-acetylation and a gain of α-(1→4)-linked glucosylation of galactose. These variants also emerged in mice at later stages of chronic infections, suggesting that they also occurred despite the animals having had time to develop an acquired immune response to the bacteria. We hypothesize that the OAg variants in invasive *S*. Typhimurium isolates from DRC might emerge as a result of immune selective pressure following secondary to multiple *S*. Typhimurium infections in a region where iNTS disease is endemic.

O-acetylation of the O:5 antigen impacts the binding of different antibodies, including various monoclonal (37), polyclonal (38), and O:4 antigen (39) antibodies. In mice immunized with an *S*. Typhimurium strain possessing an intact and functional *oafA* gene, the median antibody titer against Abe-O-acetylated LPS was 32-fold higher than the titer against de-O-acetylated LPS. Mice immunized with a strain carrying a disrupted *oafA* gene showed an 8-fold-higher titer against de-O-acetylated LPS. Thus, OAg O-acetylation can alter recognition by some antibodies targeting *S*. Typhimurium OAg (38). This differential antibody recognition of OAg had a statistically significant correlation with protection against subsequent challenge with *S*. Typhimurium *oafA*^+^ and *oafA*-deleted strains, carrying an insertion that truncated the O-acetylation (38). It has also been suggested that the three-dimensional (3D)structure of LPS can be altered upon Abe O-acetylation (37). Less is known about the role of the O-acetylation of Rha. In 2015, Lanzilao et al. (40) reported that D23580 OAg, which is O-acetylated on Rha, when conjugated to the carrier protein CRM_197_, induced antibodies that bound the homologous OAg more efficiently than OAg O-acetylated only at Abe but maintained bactericidal activity against *S*. Typhimurium isolates that expressed different OAg forms. *S*. Typhimurium OAg *O*-acetyls were confirmed to be immunodominant epitopes in more recent work by Baliban et al. (18). Those authors suggest that antibodies to O-acetylated epitopes are induced under conditions where protection is achieved and that immunization with de-O-acetylated OAg glycoconjugates provides significant but reduced protection against *S*. Typhimurium challenge.

*O*-Acetyl groups are important immune determinants for several bacterial polysaccharide vaccines (41). Konadu et al. (42) reported that *O*-acetyl groups are required in order to elicit anti-LPS antibodies with bactericidal activity against S. enterica serovar Paratyphi A. Sera from mice injected with conjugates of LPS detoxified by hydrazinolysis, which removes *O*-acetyl groups, had no detectable anti-LPS antibodies and had no bactericidal activity. Also, in S. enterica serovar Typhi LPS OAg, glucosylation and O-acetylation impacted serum resistance and antibody recognition and were hypothesized to be immune evasion mechanisms (43).

Our study highlights substantial convergent evolution within the recent *S*. Typhimurium population in the DRC toward O:5 loss, alongside O-acetylation of Rha. Importantly, our results confirmed that O:5^−^ isolates show decreased antibody binding and subsequent complement-mediated killing compared to their O:5^+^ counterparts when using a mouse monoclonal antibody. Although no information is known about the epitope recognized by this commercial monoclonal antibody, the presence of O-acetylation on Abe significantly impacts antibody recognition and killing of *S*. Typhimurium strains. The availability of other well-characterized monoclonal antibodies will allow further investigation of their ability to recognize different OAg structures. Moreover, there is no information on how a monoclonal antibody can represent the complex polyclonal response after vaccination and how antibodies elicited by O:5^+^ *S*. Typhimurium OAg can protect against circulating O:5^−^ isolates. The observed variability in OAg O-acetylation patterns among *S*. Typhimurium clinical isolates may have implications for the optimal design of OAg-based vaccines, and this aspect needs to be further explored.

## MATERIALS AND METHODS

### Ethics statement.

Ethical approval for microbiological surveillance was granted by the Institutional Review Board at the Institute of Tropical Medicine (ITM) in Antwerp, Belgium, and by the Ethics Committees of Antwerp University (Belgium) and the School of Public Health, Kinshasa, DRC.

### Study setting.

*S*. Typhimurium blood isolates from the DRC were available from two collections. The DRC is the largest country in Central Africa, and some surveillance sites are >2,000 km apart without highways connecting them. Isolates from 2002, 2005, 2006, 2007, and 2008 originating from Lwiro (Sud Kivu province, near the Rwandese border in the eastern DRC) were included in this study. These isolates had been stored at the Saint Pierre Hospital in Brussels, Belgium. Additionally, blood culture surveillance in the DRC has been organized by the teams of the National Institute of Biomedical Research (INRB) and the ITM from 2007 to 2017 at the referral hospital of Saint Luc in Kisantu (Kongo-Central province, western DRC); the University Hospital of Kinshasa, the referral Hospital St. Joseph, and Monkole Hospital in Kinshasa (Kinshasa province, western DRC); the referral hospital of Bwamanda (Sud Ubangi province, northwestern DRC); and the referral hospital of Kabondo and the University Hospital of Kisangani (CUKIS) in Kisangani (Tshopo province, northeastern DRC) (24, 44–46). Most health facilities across the country lack the capacity for diagnosing bacterial bloodstream infections, and we therefore have no information about bloodstream infections elsewhere in the DRC except as part of outbreak research (47, 48). The surveillance sites included in this study were not consistently active because of stock shortages, staff movements, and funding and security issues.

### Bacterial isolates.

In this study, 354 *S*. Typhimurium isolates were collected between 2002 and 2017 and are listed in Table S1 in the supplemental material. Twenty-eight isolates were collected between 2002 and 2008 in Lwiro in the South Kivu province of the DRC. A total of 326 isolates were collected between 2007 and 2017 in the provinces of Kongo-Central (*n* = 181), Kinshasa (*n* = 33), Sud-Ubangi (*n* = 58), Tshopo (*n* = 49), Sankuru (*n* = 1), Maniema (*n* = 1), Bas-Uele (*n* = 2), and Mai-Ndombe (*n* = 1).

All isolates were stored in tubes of Trypticase soya agar (Oxoid, Basingstoke, UK) and shipped to the ITM for confirmation and further identification. At the ITM in Belgium, isolates biochemically confirmed as Salmonella spp. were serotyped using commercial antisera (Sifin, Berlin, Germany) according to the Kauffmann-White scheme (49), including O:5 reactivity. A representative selection of the isolates (10%) was sent to the National Reference Centre Sciensano in Belgium for confirmation of serotype.

### Stability of O:5 specificity over generations.

The stability of O:5 specificity over time and generations was studied for three random O:5^−^ and three O:5^+^ isolates. The outcome would then support whether the O:5 specificity is a bistable phenotype or has an underlying genetic cause. Bacteria were repetitively inoculated onto blood agar plates and incubated overnight. Three colonies per plate were subcultured daily 12 times by inoculating them onto fresh blood agar or Mueller-Hinton (MH) agar plates. O:5 serotyping was performed after the 1st, 2nd, 3rd, 4th, 8th, and 12th inoculations.

### Illumina whole-genome sequencing and single nucleotide polymorphism analysis.

Whole-genome sequencing data were obtained as described previously (4). In brief, DNA for Illumina sequencing was extracted using the Gentra PureGene Yeast/Bact kit (Qiagen, Hilden, Germany), according to the manufacturer’s guidelines. Genomic DNA was then subjected to indexed whole-genome sequencing on an Illumina HiSeq 2500 platform at the Wellcome Sanger Institute to generate paired-end reads of 100 to 150 bp in length.

Illumina HiSeq reads were mapped to the *S*. Typhimurium reference genome of ST313 lineage II (D23580) (GenBank accession number FN424405.1) (50) using SMALT v0.7.4 to produce a BAM file. SMALT was used to index the reference using a kmer size of 20 and a step size of 13, and the reads were aligned using default parameters but with the maximum insert size set as 3 times the mean fragment size of the sequencing library. PCR duplicate reads were identified using Picard v1.92 (Broad Institute, Cambridge, MA, USA) and flagged as duplicates in the BAM file.

Variation detection was performed using samtools mpileup v0.1.19 with parameters “-d 1000 -DSugBf” and bcftools v0.1.19 (51) to produce a binary variant call format (BCF) file of all variant sites. The option to call genotypes at variant sites was passed to the bcftools call. All bases were filtered to remove those with uncertainty in the base call. The bcftools variant quality score was required to be greater than 50, and the mapping quality (map_quality) was required to be greater than 30. If not all reads gave the same base call, the allele frequency, as calculated by bcftools, was required to be either 0 for bases called the same as the reference or 1 for bases called a single nucleotide polymorphism (SNP) (af1 < 0.95). The majority base call was required to be present in at least 75% of reads mapping at the base (ratio of <0.75), and the minimum mapping depth required was 4 reads, at least 2 of which had to map to each strand (depth of <4; depth_strand of <2). Finally, strand_bias was required to be less than 0.001, map_bias was required to be less than 0.001, and tail_bias was required to be less than 0.001. If any of these filters were not met, the base was called uncertain.

### Phylogenetic analysis.

A pseudogenome was constructed by substituting the base call at each site (variant and nonvariant) in the BCF file into the reference genome, and any site called uncertain was replaced with an N. Insertions with respect to the reference genome were ignored, and deletions with respect to the reference genome were filled with N’s in the pseudogenome to keep it aligned and the same length as the reference genome used for read mapping.

Recombinant regions in the chromosome such as prophage regions and the *fljB* coding sequence (CDS) were checked using Gubbins v1.4.10 (52) and removed from the alignment. SNP sites were extracted from the alignment using snp-sites software (53) and used to construct a maximum likelihood phylogeny. RAxML v8.2.8 (54) with the substitution model GTRCAT was used. Support for nodes on the trees was assessed using 1,000 bootstrap replicates. The tree was rooted to *S*. Typhimurium ST313 strain DT2. Trees were visualized using Figtree v1.4.2 and iTOL (55).

### Pangenome analysis.

The Illumina data were *de novo* assembled using VelvetOptimiser v.2.2.5 and Velvet v1.2.10 (56). Assemblies were improved by scaffolding with the best *N*_50_ and contigs using SSPACE v2.0 (57), and sequence gaps were filled with GapFiller v1.11 (58). Assemblies were annotated using PROKKA v1.5 (59) and a Salmonella-specific database from RefSeq (59).

The pangenome of the full data set was constructed using Roary (60), with a BLASTp percent identity of 95% and a core definition of 99%. Genes significantly associated with the O:5 phenotype as determined using serotyping were identified using Scoary v.1.6.16 (60).

Conservation of the *oafA* gene across all assemblies was analyzed using BLASTN 2.6.0+ using the intact *oafA* sequence of O:5^+^ isolate 10808/3 as the query and the assemblies of all isolates as subjects.

### MinION long-read sequencing and genomic analysis.

*S*. Typhimurium isolates 18034/3, 4701/4, 6088/3, 7123/11, 11480/3, 1304, and 3832/3 were grown overnight on MH agar at 37°C. For Oxford Nanopore sequencing, genomic DNA was extracted using the Epicentre MasterPure complete DNA and RNA purification kit (Lucigen, Middleton, WI, USA). A multiplex library was prepared using the 1D ligation sequencing kit (catalog number SQK-LSK109) and the native barcode expansion kit (catalog number EXP-NBD104) (Oxford Nanopore Technologies [ONT], Oxford, UK). Sequencing was performed using the Flo-Min110 flow cell on a MinION Mk1B device. Guppy 4.0.14 (ONT, Oxford, UK) was used for base calling (fast mode) and demultiplexing. Reads were trimmed using Porechop v0.2.4 (61), and high-quality reads were filtered using Filtlong v0.2.0 (62). Hybrid *de novo* assemblies of Illumina and Nanopore reads were generated using Unicycler v0.4.8 (default parameters) (63). Easyfig v2.2.3 (64) and Inkscape v0.92 were used to create figures comparing genomic regions. Insertion sequence (IS) elements were annotated using ISfinder (65).

### Selection of isolates for physicochemical analysis.

A selection of isolates was subjected to further analysis to reveal the physicochemical structure of the OAg. O:5^+^ and O:5^−^ isolates covering the different types of *oafA* recombination events and encompassing spatiotemporal variation in the collection were selected for further analysis. All O:5^−^ isolates with intact *oafA* genes were additionally included in the analysis. In total, 11 O:5^+^ and 14 O:5^−^ isolates were subjected to physicochemical analysis.

### Physicochemical characterization.

All isolates were grown at 30°C in liquid Luria-Bertani (LB) medium overnight in a rotary shaker at 180 rpm. OAg extraction was performed directly on bacterial cells by acid hydrolysis as previously described (66). In particular, the bacterial pellet was separated by centrifugation, resuspended in 1% acetic acid, and heated at 100°C for 2 h. Supernatants containing OAg were dried, resuspended in water, and purified using a PD10 column prepacked with Sephadex G-25 Superfine (GE Healthcare, Marlborough, MA, USA). Part of the samples was lyophilized and exchanged twice with 99.9% deuterium oxide (D_2_O), dissolved in 600 μL of D_2_O, and introduced into a 5-mm nuclear magnetic resonance (NMR) tube for data acquisition. ^1^H NMR spectroscopy was used to confirm the OAg identity and determine the O-acetylation pattern (21). Spectra were recorded at 303 K with a Bruker Avance III 400 spectrometer using standard pulse sequences. ^1^H NMR spectra were recorded at 400 MHz, chemical shift values are reported in parts per million, and the solvent peak for D_2_O was calibrated at 4.70 ppm. Data acquisition and processing were performed with the TopSpin 3.5 software package (Bruker BioSpin).

High-performance anion-exchange chromatography with pulsed amperometric detection (HPAEC-PAD) was used to verify the expected OAg composition, evaluate the OAg amount (expressed as micrograms of OAg per bacterial optical density [OD] unit), and quantify the percentage of glucosylation (21).

OAg samples were also characterized by high-performance liquid chromatography–size exclusion chromatography (HPLC-SEC) with differential refractive index (dRI) detection to estimate the molecular size distribution. The OAg samples were run on a TSK gel G3000 PWXL column (30 cm by 7.8 mm, particle size of 7 μm, catalog number 808021) with a TSK gel PWXL guard column (4.0 cm by 6.0 mm, particle size of 12 μm, catalog number 808033) (Tosoh Bioscience Tokyo, Japan). The mobile phase was composed of 0.1 M NaCl, 0.1 M NaH_2_PO_4_, and 5% CH_3_CN (pH 7.2) at a flow rate of 0.5 mL/min (isocratic method for 35 min). The OAg peak molecular weight was calculated using dextrans as standards in the range of 12 to 150 kDa.

Some samples were selected for composition and linkage analysis by the use of gas-liquid chromatography coupled with mass spectrometry (GLC-MS) and ^1^H NMR analysis for quantifying the O-acetylation level. Such samples were further purified by gel filtration chromatography on a HiPrep 16/60 Sephacryl S300 HR column (600 by 16 mm; GE Healthcare, Marlborough, MA, USA), with elution with phosphate-buffered saline (PBS) at 0.5 mL/min.

For the determination of the glucosylation level of Gal, the OAg samples were subjected to linkage analysis: 0.5 mg of each sample was permethylated (67), hydrolyzed with 2 M trifluoroacetic acid at 125°C, and derivatized to alditol acetates (68). The mixtures of partially methylated alditol acetates obtained were subjected to GLC analysis on an Agilent Technologies 6850 gas chromatograph equipped with a flame ionization detector and using helium as the carrier gas. Separation was performed using an HP-1 capillary column (30 m; Agilent Technologies) with the following temperature program: 1 min at 125°C, 125°C to 240°C at 4°C/min, and 2 min at 240°C. GLC-MS analyses were carried out on an Agilent Technologies 7890A gas chromatograph coupled to an Agilent Technologies 5975C VL MSD, using the same experimental conditions as the ones described above. Integration values of the areas of the partially methylated alditol acetates were corrected by the effective carbon response factors (69).

### Antibody binding to bacteria and bactericidal activity.

Fluorescence-activated cell sorting (FACS) analysis was performed as previously described (70). Bacteria were pelleted, washed with PBS, and then blocked with PBS containing 3% (wt/vol) bovine serum albumin (BSA) for 15 min. Two commercial antibodies targeting Salmonella Typhimurium LPS were used: a mouse monoclonal antibody (catalog number ab8274; Abcam) and a rabbit polyclonal serum (anti-O:4, catalog number 294401; Denka Seiken). Bacteria were incubated with antibodies diluted in PBS plus 1% (wt/vol) BSA (1:2,000) for 1 h. After washes, samples were incubated with Alexa Fluor 647 goat anti-mouse IgG or Alexa Fluor 488 goat anti-rabbit IgG (1:500) (Molecular Probes) for 30 min. Finally, bacteria were fixed with 4% (wt/vol) formaldehyde for 20 min, and flow cytometry analysis was performed using a FACSCanto II flow cytometer (BD Biosciences).

The same antibodies were tested against the selected bacterial strains in a serum bactericidal assay (SBA) based on the luminescence readout (71), as previously described (31). The results of the assay were expressed as the 50% inhibitory concentration (IC_50_), the reciprocal serum dilution that resulted in a 50% reduction of luminescence, thus corresponding to 50% growth inhibition of the bacteria present in the assay mixture. GraphPad Prism 7 software was used for curve fitting and IC_50_ determination.

### Data availability.

Newly generated sequencing data are publicly available at the European Nucleotide Archive (study accession number PRJEB20135).
