# Supplementary figures and images for: Genetic and Structural Variation in the O-Antigen of Salmonella enterica Serovar Typhimurium Isolates Causing Bloodstream Infections in the Democratic Republic of the Congo
Source: mBio. 2022 Jul 18;13(4):e00374-22. doi: 10.1128/mbio.00374-22 (PMC9426603; doi:10.1128/mbio.00374-22)

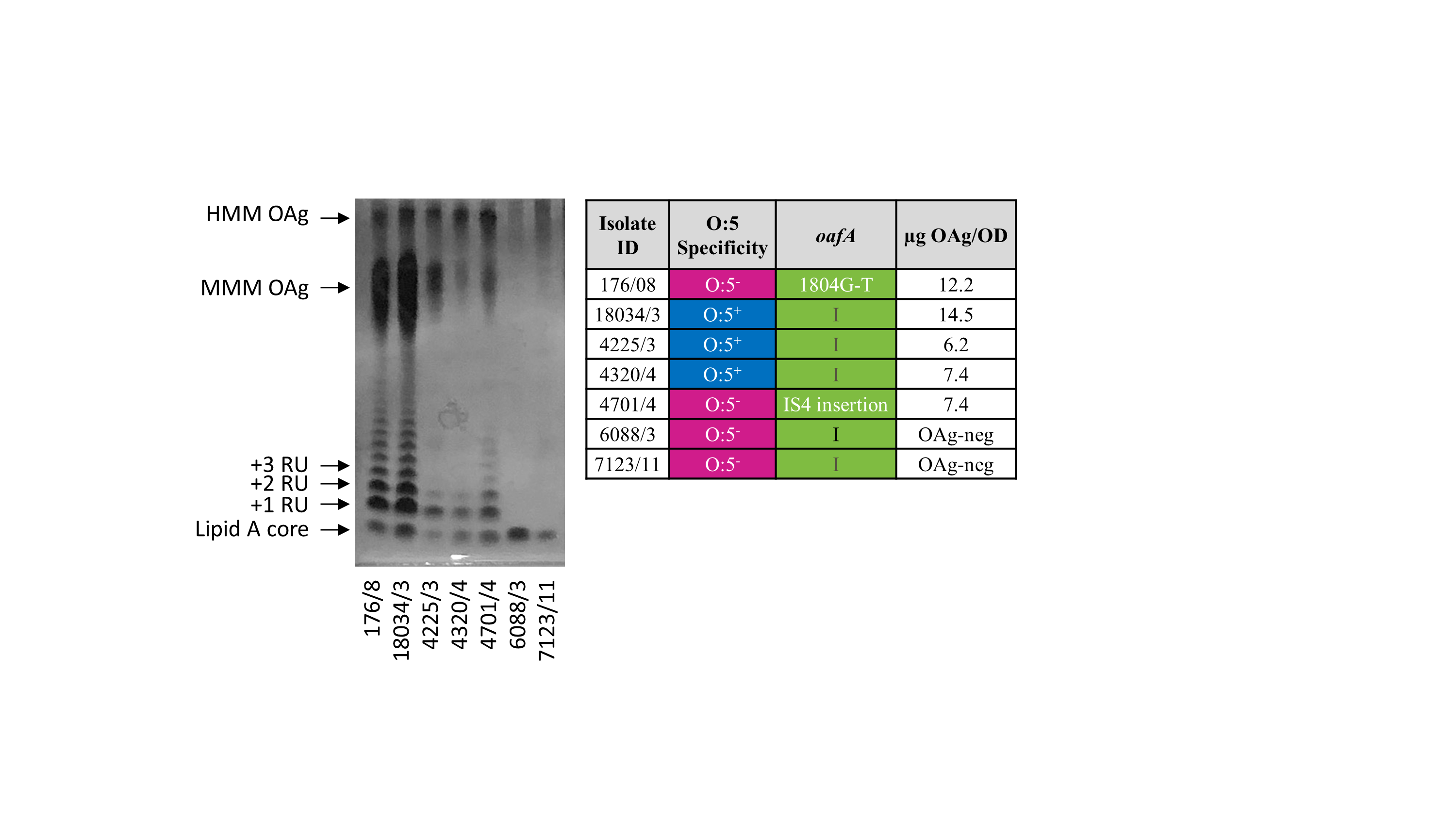

Supplement: FIG S2 [file mbio.00374-22-s0003.tif]
