# Supplementary material for: Genetic and Structural Variation in the O-Antigen of Salmonella enterica Serovar Typhimurium Isolates Causing Bloodstream Infections in the Democratic Republic of the Congo
Source: mBio. 2022 Jul 18;13(4):e00374-22. doi: 10.1128/mbio.00374-22 (PMC9426603; doi:10.1128/mbio.00374-22)
Supplement: TABLE S2 [file mbio.00374-22-s0004.docx]

**Supplementary Table 2:**

| **Isolate ID** | **% O-acetylation** | | |
| --- | --- | --- | --- |
|  | **% 3/2 OAc Rha** | **% 2 OAc Abe** | **Tot % OAc** |
| 18034/3 | - | 56 | 56 |
| 8128/12 | 66 | - | 66 |
| 8692/3 | 75 | - | 75 |
| 13404/3 | 86 | 68 | 154 |
| 10328/3 | 84 | 41 | 125 |
