# Supplementary material for: Genetic and Structural Variation in the O-Antigen of Salmonella enterica Serovar Typhimurium Isolates Causing Bloodstream Infections in the Democratic Republic of the Congo
Source: mBio. 2022 Jul 18;13(4):e00374-22. doi: 10.1128/mbio.00374-22 (PMC9426603; doi:10.1128/mbio.00374-22)
Supplement: TABLE S3 [file mbio.00374-22-s0005.docx]

**Supplementary Table 3:**

| **Isolate ID** | **% α1-4 glucosylation** |
| --- | --- |
|  |  |
| 18034/3 | 32 |
| 8128/12 | 48 |
| 8692/3 | 11 |
| 13404/3 | 13 |
| 10328/3 | 52 |
